# Maternal–fetal attachment in pregnant Italian women: multidimensional influences and the association with maternal caregiving in the infant’s first year of life

**DOI:** 10.1186/s12884-021-03964-6

**Published:** 2021-07-06

**Authors:** Chiara Sacchi, Marina Miscioscia, Silvia Visentin, Alessandra Simonelli

**Affiliations:** 1 Department of Developmental and Social Psychology, University of Padova, via Venezia 8, Padova, Italy; 2 Division of Women’s and Child’s Health, University of Padova, Padova, Italy

**Keywords:** Pregnancy, Maternal–fetal attachment, Mental health, Caregiving

## Abstract

**Background:**

Maternal–Fetal Attachment (MFA) describes the cognitive-representational, emotional, and behavioral aspects of the mother–fetus relationship that develops during pregnancy.

We present two studies conducted on pregnant Italian women. In Study I, we aimed to explore multifaceted associations of MFA with variables important for a healthy pregnancy (e.g., maternal mental health, the couple’s relationship). In Study II, we investigated the predictive role of MFA on observed maternal caregiving during the first months of the infant’s life.

**Methods:**

In Study I, 113 pregnant Italian women were assessed on MFA (*Maternal Antenatal Attachment Scale*, MAAS), maternal depression (*Beck Depression Inventory-II*, BDI-II), maternal anxiety (*State Trait Anxiety Inventory – State version,* STAI), adjustment of the couple (*Dyadic Adjustment Scale,* DAS), and perceived parental care (*The Parental Bonding Instrument,* PBI). In Study II, 29 mother–infant pairs were followed up at 4 months to assess observational variables of maternal caregiving through the *Emotional Availability Scale* (EAS) and to test for an association with MFA in pregnancy.

**Results:**

Study I showed a significant association between MFA and the quality of the couple relationship (*β* = .49, *P *< .001) and between MFA and the recall of memories of care received in childhood (*β* = .22, *P *= .025). Study II showed a predictive effect of MFA on maternal structuring observed during mother–infant interactions at 4 months of age (*β* = 0.36, *P *= .046).

**Conclusion:**

The study points out relevant relationship contexts that might receive care and support throughout pregnancy to protect MFA. The findings also provide thoughtful insights on the role of MFA in early maternal caregiving, suggesting that MFA might be a candidate as one putative antecedent of mother–infant interaction processes.

## Background

Parenting begins before birth [1]. A substantial body of research has identified pregnancy as a valuable window for maternal and fetal health [2, 3], as well as for the emergence of key determinants of parenting and the mother–infant relationship [4, 5]. This window of plasticity provides the opportunity to deliver timely programs to support expectant parents [6], but it also requires identification of the prenatal influences that promote mother–fetus bonding during pregnancy and an understanding of how expectant mothers’ emotional bonds with the their unborn infants might contribute to later variation in their caregiving practices.

The maternal–fetal attachment (MFA) was introduced for the first time in 1981 by Cranley [7] to describe the emotional bond that an expectant mother develops towards her unborn baby [8]. MFA involves maternal engagement in positive prenatal health practices and behaviors conveying caring, commitment, and interaction with the unborn child, thus emphasizing the establishment of a unique prenatal relationship [7, 9]. Several descriptions have been proposed for MFA, suggesting that the concept has a multi-dimensional nature encompassing thoughts, feelings, and dispositions to protect, interact with, and meet the needs of the fetus [7, 10, 11]. Such a prenatal bond seems to anticipate the key ingredients of the post-natal caregiving system [12], which comprises the child’s protection, comfort, and care, through parental perception and responsiveness to child’s signals, support to the exploration, and the provision of comfort in times of distress [13, 14]. Studies on maternal caregiving have underscored that the experience of a connection with the unborn child and the maternal mental representation of the fetus might set the cognitive-representational, emotional, and behavioral bases of the post-natal mother–child relationship [15]. The limited existing studies investigating the association between MFA and maternal caregiving in first years of life support a positive association of MFA with maternal sensitivity and a mother’s responsiveness to her infant [16]. However, when maternal caregiving through direct observations of mother–infant free play interactions were assessed, no significant association between MFA and maternal care were found [17], suggesting that the extant research on MFA and maternal caregiving provide findings limited by parent-reported assessment of caregiving quality and leaves the role of MFA in observable caregiving practices as an open question.

Along with the need to understand the importance of MFA in postnatal caregiving, it is of clinical relevance to identify the key influences of MFA during pregnancy to direct prenatal intervention strategies aimed at protecting and supporting MFA. So far, studies investigating the characteristics of pregnancy associated with MFA have largely suggested mutual influences between MFA and maternal mental health [18], with negative associations reported for depressive symptoms [19, 20] and anxiety [21]. The complex physical and psychological upheavals of pregnancy and the multidimensional nature of MFA still suggest further exploration of additional prenatal influences. So far, social support has been found to play a moderate role in MFA, while socio-demographic characteristics, such as parity, age, marital status, income, and education, showed weaker associations with MFA levels [22, 23]. Largely underexplored remain the relational influences in pregnancy, such as the relationship of the expectant mother with her own mother and the couple’s marital relationship. A recent review has shown that the couple’s relationship potentially affects MFA, with a positive association between a good intimate partner relationship and higher MFA [12]. In addition, throughout pregnancy, the internal representation of the relationship with the expectant mother’s own mother is progressively activated and reworked in the mind of the expectant mother to build her own new parental attitudes [24]. Unfortunately, despite such theoretical consideration and the great importance that both couple and parental relationships have during pregnancy [25], no study has attempted to explore the role of memories of received maternal care in childhood in expectant mothers’ MFA, and only very few have investigated the role of the couple’s relationship in MFA.

Overall, studies have underscored the importance of the prenatal mother–fetus bond in maternal caregiving and the establishment of a secure mother–child relationship [26]; however, studies on MFA and observed maternal caregiving are lacking. In addition, given the potential role of MFA in maternal caregiving and considering the multidimensional influences of MFA, more intensive investigations of the influences of MFA in pregnancy are needed, including a focus on the influences of relationships.

To this end, we present two studies conducted at the Department of Women’s and Childs’s Health of the Padua University Hospital and the Department of Developmental and Social Psychology of the University of Padua, Italy. The studies aimed to address important questions about the influences of MFA and its role in maternal caregiving. Study I investigated the association between MFA and several characteristics of the third trimester of pregnancy. We investigate the association between MFA, mental health indexes, and relational functioning, under the hypothesis that MFA is negatively associated with mental health symptoms (i.e., depression or anxiety) and positively associated with both the quality of couple adjustment and the recall of memories of received care in childhood. Study II is aimed at exploring the longitudinal association between third-trimester MFA and the quality of observed caregiving during free-play interactions. Here, we expect that MFA is positively associated with better caregiving qualities (i.e., maternal sensitivity, maternal structuring) observed at the fourth month of the infant’s life.

## Methods

### Participants

The studies were conducted at the Department of Women’s and Childs’s Health of the University Hospital of Padua and at the Department of Developmental and Social Psychology of the University of Padua, Italy.

Pregnant women in their third trimester residing in northern-east Italy and attending the Padua University Hospital (Padua, Italy) for obstetric and gynecological visits were recruited. The exclusion criteria were as follows: i) age < 18 years, ii) risk of psychiatric disorders as defined by clinical score (namely a Global Symptom Index > 65) in the Symptom Checklist 90-Revised [27], iii) insufficient Italian language proficiency, especially in reading and comprehending the self-report measures, iv) preterm delivery (< 37 weeks of gestation). A total of N = 127 pregnant women took part voluntarily in Study I; a subgroup of participants (N = 29) also agreed to be contacted for Study II, which took place after delivery.

### Procedure

Pregnant women were recruited from prenatal classes and/or immediately after echography visits to the Obstetrics and Gynecological Clinic of Padua University Hospital and were invited to participate in Study I: participants who voluntarily agreed to participate in the research were invited to complete a series of validated self-report questionnaires after attending the obstetric and gynecological visit. The assessment was performed at the clinic in a quiet room, in the presence of a psychologist-in-training, who was there to answer questions and address any doubts about the research and methods. Following completion of Study I, Study II was briefly presented so that participants could decide whether to give their consent to be contacted after delivery for Study II. Participants available for Study II were contacted by telephone and were invited to visit the Laboratories of the Department of Developmental and Social Psychology of the University of Padua (Padua, Italy) for an observational assessment, as part of a broader longitudinal study [28]. This assessment was conducted when the child was 4 months of age and consisted of recording short sequences [10–15 min] of mother–infant free play interactions. For this assessment, a quiet room with age-appropriate toys was made available.

Both studies received ethical approval from the Institutional Review Board of the University of Padua, and all participants signed a written informed consent prior to enrollment. Data were collected between January 2018 and June 2019.

### Measures

#### Maternal–Fetal Attachment (MFA)

The *Maternal Antenatal Attachment Scale* (MAAS) [10, 29] was used to asses MFA. It is a 19-item scale describing feelings and thoughts related to the expected child. Items are scored on a 5-point Likert scale (from 1 to 5), and the sum of all items gives a total score for maternal antenatal attachment. Two subscales are also available: the quality of mother–fetus attachment (QA) and the intensity of maternal preoccupation (MP). A test for internal consistency resulted in α = 0.72.

#### Maternal mental health in pregnancy

Maternal mental health during pregnancy was assessed in terms of symptoms of depression and/or anxiety. Depression was investigated through the *Beck Depression Inventory-II* (BDI-II) [30], a revised 21-item test with four response options per item (absence of a symptom [0] to severe or persistent expression of that symptom [3]). The respondent is asked to choose the statement that best reflects the way he/she has been feeling over the course of the last 2 weeks. A test for internal consistency resulted in α = 0.80. Anxiety was assessed through the *State Trait Anxiety Inventory – State version* (STAI) [31], a 20-item self-report questionnaire scored on a 4-point scale (1 = Not at all to 4 = Very much so). The state of anxiety refers to the experience of anxiety and tension at the time the questionnaire was completed (i.e., how the subject feels ‘now’). The total score (20–80) is given by the row sum of responses to each item, with higher scores indicating a greater state of anxiety. A test for internal consistency resulted in α = 0.93.

#### Couple relationship

The *Dyadic Adjustment Scale* (DAS) [32] was administered to assess the quality of the marital couple relationship. It is a 32-item self-report questionnaire assessing the degree of couple adjustment perceived by each partner. The sum of the 32 items (scored from 0 = Never agree to 5 = Always agree) gives a total score, which indicates the individual’s perceived couple adjustment, and higher scores represent greater adjustment. Cronbach’s α = 0.85 indicated a good internal consistency in this sample.

#### Maternal care received

*The Parental Bonding Instrument* – *PBI Mother Form* [33] is a 25-item scale measuring an individual’s retrospective perceptions of the parenting they received over the first 16 years of life. The questionnaire comprises two independent sub-scales: “Care” (12 items) and “Overprotection” (13 items). Items are scored on a 4-point scale ranging from 0 = Not at all to 3 = Very much. For the purpose of this study, the PBI-Mother Care and Over-protection scales were selected in order to study the recollection of memories about maternal caregiving.

Demographic information was collected, including mother’s age, level of education, marital status, and clinical information regarding the pregnancy.

#### Maternal caregiving

The *Emotional Availability Scales* (EAS) [34] coding system was applied to code maternal caregiving. Maternal caregiving in the context of parent–infant interactions was coded along four dimensions, namely maternal sensitivity, maternal structuring, maternal non-intrusiveness, and maternal non-hostility. Maternal sensitivity refers to clarity of perception and appropriate responsiveness to the child’s emotional expressions as well as maternal verbal and non-verbal emotional expressions, including positive, genuine and authentic affects; Maternal structuring conveys the parent’s ability to guide and scaffold the child’s exploration while maintaining emotional contact and availability; Maternal non-intrusiveness refers to general lack of over-direction, over-stimulation, over-protection, and intrusion while interacting with the child; maternal non-hostility describes the general absence of hostile responses, ranging from cover hostility (i.e., impatience, boredom) to openly hostile responses (i.e., raising the voice, becoming frightening). Each EA dimension produces score on a 7-point scale, where higher ratings standing for more optimal features. The quality of each maternal dimensions was evaluated during video-recorded free-play exchanges of mother–infant interactions when the child was 4 months of age.

### Analysis plan

In Study I, correlation analyses were performed to test for associations of MFA with a series of variables of interest, such as maternal mental health in pregnancy (i.e., depression and anxiety), maternal perceived maternal care, and couple adjustment. Multivariable linear regressions were performed to test the associations among the abovementioned variables, accounting for sociodemographic (i.e., age, education level, marital status) and pregnancy-related (primiparous vs multiparous) confounding effects. In Study II, multivariable linear regression analyses were performed with maternal sensitivity, maternal structuring, maternal non-intrusiveness, and maternal non-hostility as dependent variables to test for the longitudinal association between MFA and observable dimensions of maternal caregiving during interaction at 4 months of infant life. Again, maternal socio-demographic (i.e., age, education level, marital status) and pregnancy-related characteristics (primiparous vs multiparous) were used as covariates.

In all regression analyses, one index of maternal mental health in pregnancy (either depression or anxiety) was included to avoid multicollinearity effects. The interpretation of significant effects remained unaltered; the reported results refer to models accounting for the effect of depression. As an additional control, regression analyses were performed adding nationality and employment status as extra confounding variables, and no changes in the main findings were observed. Data analyses were performed using the open-source software R [35]. Two-tailed P values were used, and results were considered significant at *P *< 0.05.

## Results

### Study I

A total of 127 expectant women (mean gestational age = 36.20 weeks; standard deviation [SD] = 3.46) were recruited for Study I. Fourteen participants were excluded according to the study criteria or for missing information exceeding 10% of responses in at least one measure. Table 1 reports the demographic characteristics of the participants in Study I. Table 2 shows descriptive statistics and correlation analysis between the study variables.

**Table 1:** Study I participants’ characteristics (*N* = *113*)

*Maternal characteristics*	
Age (years)	34.29 ± 4.73
Nationality (Italian)	96 (85%)
Employment status (employed)	99 (88%)
Education	
- Lower secondary education	7 (6%)
- Upper secondary education	31 (27%)
- Bachelor’s	22 (20%)
- Master’s	38 (34%)
- Doctoral	15 (13%)
Marital status	
- Common-law partner	7 (6%)
- Co-habiting common-law partner	32 (28%)
- Married	71 (63%)
- Separated	1 (0.9%)
Parity (1st pregnancy)	62 (55%)

*Note*. Data are given as mean ± SD; No. (%)

**Table 2:** Descriptive statistics and correlations (95% confidence intervals)

		*Range*	1	2	3	4	5
1. MAAS	78.01 ± 5.30	64–90					
2. BDI	7.56 ± 4.47	0–22	-.13 (-.30, .06)				
3. STAI	39.44 ± 7.08	27–61	-.20* (-.37, -.01)	.49** (.33, .62)			
4. PBI Care	27.89 ± 6.21	6–36	.20* (.01, .37)	-.17 (-.35, .01)	-.08 (-.27, .10)		
5. PBI Over-protection	15.66 ± 5.23	5–29	-.09 (-.27, .10)	.26** (.08, .42)	.15 (-.03, .33)	-.43** (-.57, -.27)	
6. DAS	127.58 ± 11.80	61–145	.42** (.26, .56)	-.47** (-.56, -.26)	-.42** (-.56, -.26)	.12 (-.06, .30)	-.33** (-.49, -.16)

*Note*. Data are given as mean ± SD

^**^P < *.*01; * *P* < .05

*MAAS* Maternal Antenatal Attachment Scale (Condon, 1993) [10], *BDI* Beck Depression Inventory (Beck et al., 1996) [30], *STAI* State Anxiety Trait Inventory (Spielberger, 1968) [31], *PBI* Parental Bonding Instrument (Parker et al., 1989) [33], *DAS* Dyadic Adjustment Scale (Spanier et al., 1976) [32]

To assess the association of MFA with maternal mental health (anxiety or depression), the couple relationship, and perceived parental care, accounting for maternal socio-demographic (i.e., age, education, marital status), and pregnancy-related characteristics (primiparous vs multiparous), multivariable linear regression analysis was performed. Regression analysis revealed significant positive associations between MFA and the quality of couple adjustment (*β* = 0.49 *t* (100) = 4.88, *P *< 0.001) and between MFA and the perceived level of maternal care received (*β* = 0.22, t (100) = 2.28, *P *= 0.025).

### Study II

A total of 29 mothers from Study I took part in Study II; their characteristics are presented in Table 3. There were no significant differences between this sample and the women who only participated in Study I, except for a slight difference in levels of MAAS in pregnancy: Study II participants showed lower levels of MFA (*p* = 0.005), compared with Study I participants who did not agree to participate in Study II.

**Table 3:** Study II participants’ characteristics (*N* = *29*)

*Maternal characteristics*	
Age (years)	33.57 ± 4.58
Nationality (Italian)	29 (100%)
Employment status (employed)	28 (97%)
Education	
- Lower secondary education	-
- Upper secondary education	6 (21%)
- Bachelor’s	6 (21%)
- Master’s	11 (38%)
- Doctoral	6 (21%)
Marital status	
- Common-law partner	-
- Co-habiting common-law partner	7 (24%)
- Married	22 (76%)
- Separated	**-**
Parity (1st pregnancy)	13 (43%)

Note. Data are given as mean ± SD; No. (%)

The results of linear regression analyses to assess the longitudinal association of MFA with the quality of maternal caregiving in mother–infant interactions, accounting for maternal socio-demographic variables (i.e., age, education, marital status), pregnancy-related characteristics (primiparous vs multiparous), the quality of couple adjustment, the perceived level of maternal care received, and the mother’s mental health in pregnancy, are reported in Table 4.

**Table 4:** Multiple regression models for MFA on maternal caregiving dimensions (*N* = 29)

	Model 1 Maternal Sensitivity				Model 2 Maternal Structuring				Model 3 Maternal Non-Intrusiveness				Model 4 Maternal Non-Hostility			
	**b**	**SE**	***t***	***p***	**b**	**SE**	***t***	***p***	**b**	**SE**	***t***	***p***	**b**	**SE**	***t***	***p***
Intercept	0.058	5.235	-1.557	.14	-9.974	4.555	-2.189	.043	7.239	6.34	1.142	.27	4.121	3.817	1.082	.29
Mother age	0.033	0.049	0.672	.51	0.024	0.042	0.572	.57	0.068	0.059	1.148	.27	0.006	0.035	0.171	.87
Education	0.292	0.179	1.627	.12	0.324	0.155	2.083	.051	0.047	0.217	0.218	.83	0.265	0.130	2.037	.06
Marital status	0.204	0.349	0.584	.57	-0.224	0.304	-0.737	.47	-0.736	0.423	-1.741	.09	-0.376	0.254	-1.479	.15
First pregnancy	0.375	0.357	1.050	.31	0.422	0.310	1.359	.19	-0.353	0.433	-0.817	.42	0.379	0.259	1.459	.16
MAAS	0.036	0.035	1.000	.33	0.066	0.031	2.128	.046	-0.035	0.044	-0.812	.43	0.008	0.026	0.324	.75
BDI	-0.036	0.041	-0.883	.39	-0.010	0.035	-0.283	.78	-0.074	0.049	-1.495	.15	-0.057	0.030	-1.895	.07
PBI Care	0.032	0.031	1.024	.32	0.021	0.026	0.777	.45	0.008	0.037	0.218	.83	0.009	0.022	0.401	.69
PBI Over-protection	0.182	0.041	4.436	.000	0.154	0.035	4.297	.001	-0.013	0.049	-0.257	.80	0.085	0.026	2.854	.01
DAS	0.030	0.022	1.379		0.037	0.019	2.014	.068	0.004	0.026	0.145	.88	-0.003	0.016	-0.234	.83
R^2^	R^2^ = .57				R^2^ = .65				R^2^ = .38				R^2^ = .56			
F	*F*(9,19) = 2.84, *p* = .026				*F*(9,19) = 3.89, *p* = .007				*F*(9,19) = 1.28, *p* = .31				*F*(9,19) = 2.65, *p* = .035			

*Note*: *MAAS* Maternal Antenatal Attachment Scale (Condon, 1993) [10], *BDI* Beck Depression Inventory (Beck et al., 1996) [30], *PBI* Parental Bonding Instrument (PBI; Parker et al., 1979), *DAS* Dyadic Adjustment Scale (Spanier et al., 1976) [32]

## Discussion

So far, the role of maternal–fetal attachment in the quality of observed maternal caregiving has been under-investigated. This study adds to the literature in the field by highlighting: *i)* the multidimensional characteristics associated with MFA during pregnancy; *ii)* the predictive contribution of MFA to the quality of maternal caregiving observed when the infant is 4 months of age.

Study I evidenced that MFA was associated with the quality of the current romantic relationship and the levels of perceived maternal care in childhood. These findings support previous evidence showing the relational pillars of MFA [12] and confirm the importance of the couple relationship in the establishment of a healthy parent–baby relationship across the transition to parenthood [25]. Moreover, they provide the first evidence for the theoretically rooted association of MFA with the recall of memories of maternal care received in childhood. In Study I, MFA was also negatively associated with maternal anxiety during pregnancy; however, the association was no longer significant when for confounding factors were accounted for. This might be due to the low levels of maternal mental health difficulties in our sample and suggests further investigation of such contributions in more at-risk and clinical samples. Overall, Study I supports the multifaceted nature of MFA. Specifically, it gives an overview that instances of past and current relationships are active in the minds of pregnant women, perhaps modelling the maternal representations of the incoming relationship with the unborn baby.

The results of Study II show that MFA displays a positive predictive effect on maternal structuring as observed independently from behavioral mother–infant interactive exchanges at 4 months. No significant association emerged between MFA and maternal sensitivity, non-intrusiveness, or non-hostility. The specificity of the association between MFA and maternal structuring is highly important given the need for research and clinical reasons to detail the facets of the mother–infant relationship beyond the comprehensive conceptualization of maternal sensitivity. Structuring conveys the parent’s ability to guide and scaffold the child’s exploration while maintaining emotional contact and availability [34]. This ability to construct a playful setting in a way that is attuned to and will be well received by the child might tap in to emotional and cognitive and anticipatory abilities [36]. Indeed, to efficiently scaffold the interaction, parents are required to bear in mind the infant’s competencies, anticipate infant’s intentions in plausible scenarios, and control certain features of the environment in order to allow the infant to participate in aspects of play in a protected fashion. We speculate that MFA might rapidly differentiate such mothers that are already available to reflect upon their unborn baby’s signals and perhaps will be more willing to maintain such an attitude beyond the child’s birth, engaging in reflective thinking upon the infants’ intentions, capacities, affects, and thoughts while playing. Moreover, it has been observed that long-term, maternal structuring is a predictor of maternal self-esteem and the mother’s representations of herself as a parent [37]. It might be that such a process of parental identity construction finds its first steps in pregnancy, with MFA representing the first expression. That would also account for the findings of Study I, where we have observed that MFA is influenced by maternal memories of caregiving experiences in childhood, which are known to sustain maternal caregiving and infant attachment [38]. Overall, despite the limited generalizability of these findings, they validate the role of MFA in postnatal maternal care features, with the advantage of overcoming the limitation of maternal caregiving self-report evaluation. Hence, they point to the importance of prenatal maternal attachment toward the fetus as a healthy base for caregiving behaviors.

This study had some limitations. The use of self-report questionnaires in Study I should be mentioned, as it might have biased participants’ reporting on maternal mental health problems. In addition, the sample size of Study II was relatively low; this prevented us from drawing definitive and generalizable conclusion on the role of MFA in early maternal caregiving capacities and suggests the need to investigate this research question in more depth. Larger longitudinal and multi-method studies in this field are encouraged, also accounting for clinical samples, which would enable a more in-depth analysis of the association between maternal mental health in pregnancy and MFA.

## Conclusion

In a novel contribution, the present study provides thoughtful insights on the potential role of MFA as a prenatal component of maternal caregiving. The importance of such findings is given by the possibility to observe in MFA the putative prenatal antecedents of such processes of mother–infant interactions that build emotion regulation and infant mental health [39]. This assumption needs to be corroborated by further studies in order to clearly delineate the continuity of prenatal-to-postnatal parenting. In addition, we identified mental health and relational features that might receive care and sustain throughout pregnancy to protect MFA and its positive effects on infant development across the transition to parenthood. Further studies ought to follow-up infants in the first years of life to explore different nuances of the early mother–infant relationship sustained by MFA.

## Data Availability

All data and materials support the study claims and comply with field standards. The datasets used and/or analyzed during the current study are available from the corresponding author on reasonable request.

## Abbreviation

MFA: Maternal-fetal attachment
